# LiDAR Point Cloud Data Combined Structural Analysis Based on Strong Form Meshless Method Using Essential Boundary Condition Capturing

**DOI:** 10.3390/s23136063

**Published:** 2023-06-30

**Authors:** Kyung-Wan Seo, Young-Cheol Yoon, Sang-Ho Lee

**Affiliations:** 1 Department of Civil and Environmental Engineering, Yonsei University, Seoul 03722, Republic of Korea; k.w.seo@yonsei.ac.kr; 2 Department of Civil Engineering, Myongji College, Seoul 03656, Republic of Korea

**Keywords:** light detection and ranging, point cloud data, particle difference method, essential boundary condition, structural monitoring

## Abstract

This study proposes a novel hybrid simulation technique for analyzing structural deformation and stress using light detection and ranging (LiDAR)-scanned point cloud data (PCD) and polynomial regression processing. The method estimates the edge and corner points of the deformed structure from the PCD. It transforms into a Dirichlet boundary condition for the numerical simulation using the particle difference method (PDM), which utilizes nodes only based on the strong formulation, and it is advantageous for handling essential boundaries and nodal rearrangement, including node generation and deletion between analysis steps. Unlike previous studies, which relied on digital images with attached targets, this research uses PCD acquired through LiDAR scanning during the loading process without any target. Essential boundary condition implementation naturally builds a boundary value problem for the PDM simulation. The developed hybrid simulation technique was validated through an elastic beam problem and a three-point bending test on a rubber beam. The results were compared with those of ANSYS analysis, showing that the technique accurately approximates the deformed edge shape leading to accurate stress calculations. The accuracy improved when using a linear strain model and increasing the number of PDM model nodes. Additionally, the error that occurred during PCD processing and edge point extraction was affected by the order of polynomial regression equation. The simulation technique offers advantages in cases where linking numerical analysis with digital images is challenging and when direct mechanical gauge measurement is difficult. In addition, it has potential applications in structural health monitoring and smart construction involving machine leading techniques.

## 1. Introduction

Evaluating the usability of a target structure and verifying whether it meets the required structural performance necessitates obtaining kinematic variables such as deformation, strain, and stress. Typically, these variables are measured using contact sensors or non-contact measurement equipment that tracks installed targets [1,2]. Contact sensor-based methods allow continuous real-time responses, making tracking strain or displacement at measurement points easy. However, these methods have drawbacks, such as requiring significant resources to process accumulated data and difficulty attaching sensors to actual structures [3]. Moreover, recent advancements in the resolution, accuracy, and usability of remote sensing devices, such as digital cameras and light detection and ranging (LiDAR), have enabled the development and application of methods that can complement the limitations of traditional contact measurement devices [4] and are being researched and implemented for better understanding and capturing of a real-world problem [5].

Although images can provide perspective data of a subject, they require additional processing to be used as coordinate or location information in planes or spaces. In contrast, LiDAR can provide point cloud data (PCD), a group of three-dimensional points representing the surface of a reflecting object, by measuring the time of flight of laser pulses and instantaneous scanning angles. Ongoing efforts are being made to apply this data for monitoring and analyzing target objects. For example, a method was proposed to compare PCD from different time points to identify damaged areas based on an initial reference and update the analysis model [6]. Additionally, research was published that estimates structural displacement using the Hausdorff distance concept calculated from PCD acquired at different time points [7,8,9].

However, although effective in capturing overall trends, the displacement tracking method can result in significant errors when reproducing overall kinematic variables based on the random distribution of points. Alternatively, some methods use polynomial regression to estimate overall displacement and strain by approximating the targets’ surface or cross-sectional shape in the PCD. Cabaleiro et al. [10] proposed a method for modeling a deformed beam using PCD acquired from a steel beam subjected to bending and torsion. They used polynomial surface fitting to model the deformation of the beam and demonstrated that it is possible to automate the process of analyzing whether the maximum limits specified in structural codes are exceeded by comparing them with idealized straight beams. However, although deflections from the initial straight beam could be determined, no attempts were made to calculate or extract displacements, and therefore strain and stress were not estimated.

Jo et al. [11] and Jo et al. [12] demonstrated that it is possible to approximate displacements across an entire area by approximating the PCD cross-section of a curved steel plate subjected to lateral pressure with a polynomial function and creating a 3D grid model for the entire surface using kriging. This method has the advantage of being applicable even in cases where no measurement targets are available. However, it is limited by the fact that the cross-sectional curvature is not significant because of the structural features designed to withstand lateral pressure, and the displacement model depends on the grid, making it challenging to apply when boundaries change. In [13], curve coefficients for a quadratic polynomial function approximating the surface of a deformed beam were estimated using a genetic algorithm. This enabled the determination of the deflection of the entire beam, such that the deflection value at the center could be calculated with high accuracy. However, this method is computationally complex and requires high computational costs because of using a genetic algorithm, and the analysis focuses on deflection calculation rather than kinematic and kinetic variables such as strain and stress.

In the past, research has primarily utilized methods that combine the finite element method (FEM) with non-contact sensors such as digital cameras or LiDAR to reproduce kinematic variables at desired locations from displacement information acquired directly or indirectly at arbitrary positions [14,15,16]. Generally, FEM meshes and measurement points are aligned to calculate displacements at arbitrary positions numerically, and the tracked data of measurement point changes are processed and reflected in the FE nodes. If the numerical analysis model requires displacement at a non-node location, strain, or stress at a non-quadrature point, interpolation or extrapolation can be utilized. However, in combination with FEM, it is necessary to reconstruct the mesh at each time point when the deformation tracking simulation of the target structure is performed, and mesh reconstruction becomes more challenging when the target formation is complex or when discontinuities, such as cracks, are involved.

To avoid the difficulties associated with using meshes in numerical analysis using PCD, techniques such as the boundary element method (BEM) [17] or the element-free Galerkin method (EFGM) [18] can be considered. However, using the BEM requires finding boundary values and calculating kinematic variables using a fundamental solution. This can be challenging with limited measurement values based on an experiment. While the EFGM is a meshless technique, it still requires integration cells similar to meshes to calculate integrals for weak formulations during problem solving. Thus, it cannot be considered entirely free from mesh constraints [19,20,21]. Furthermore, the EFGM approximation function requires burdensome differentiation calculations, which are computationally expensive for variables such as strain or stress.

To overcome the limitations of numerical analysis using PCD with meshes, a meshless technique that enables fast differentiation calculations and is not constrained by mesh or grid construction is necessary for calculating kinematic variables. The particle difference method (PDM) is a meshless technique that uses strong form or collocation-based rapid derivative approximations to quickly calculate displacement, strain, or stress without the meshes, grids, or integration cell. Since the modeling is performed using only nodes, any changes in the geometric shape of the target structure over time do not pose additional difficulties [22,23,24,25]. Godinho et al. [26] presented a structural analysis method that combines digital image processing (DIP) and the EFGM. It predicted crack patterns by measuring target point displacements using DIP and computed stress using a meshfree approximation based on these displacement values. However, coupling DIP techniques with meshless methods, as demonstrated by Godinho et al. [26], might be challenging. While meshless approximation functions are used in this approach, combining advanced DIP with numerical techniques for solid mechanics problem solving is still limited.

Park et al. [27] investigated the combination of DIP and the PDM for simulating real-scale tests by calculating kinematic variables using displacement information obtained from digital images taken during the loading process of a structure at attached targets. The advantage of the PDM, which can adapt to changing node numbers during analysis, was utilized to increase the number of tracking points and improve the accuracy of numerical analysis results. However, this method relies on the attachment of targets to the target structure and tracking their displacements, which might make it impractical in situations where such attachments are not possible.

This study presents a novel method for determining kinematic variables across the entire area of a loaded structure by utilizing pre- and post-deformation PCD obtained through LiDAR scanning of a target-free structure and applying it to a strong form-based meshless method. This method estimates structural deformation by analyzing the PCD, extracting edge shape changes, and assigning essential boundary conditions to the numerical model boundary for the target structure. The governing differential equations of solid mechanics are given as a form of boundary value problem (BVP). A Dirichlet boundary problem is mainly considered here since the captured deformation is assigned as the essential boundary condition. The shape of the deformed structure is expressed through LiDAR PCD, edge extraction, and polynomial regression, and the Dirichlet boundary problem solving is conducted consecutively. Edge extraction was performed using eigenvalues for k-nearest neighbors in the acquired PCD. Since the PDM is the strong form-based meshless technique, creating a numerical model and handling the essential boundary condition is straightforward once the boundary displacements are determined by analyzing the PCD, allowing for rapid stress calculation in the target structure.

In addition, the PDM has no issues even when the number of nodes increases owing to changes in node positions or changes in the analysis steps. Instead, we conducted an error analysis to validate the proposed method by applying it to a problem with a known analytic solution. Moreover, we demonstrated the method’s robustness by performing various simulations of a three-point bending test for a rubber beam and suggested several approaches to ensure accurate simulation results.

## 2. Formulation of Boundary Value Problem for PDM

### 2.1. Derivative Approximation

The PDM is a meshless method that uses a strong formulation to analyze the stress of the target structure using nodes only. Governing equations are discretized directly at each node to obtain the numerical solution. The derivative approximation required for this process is obtained by constructing a Taylor series expansion for the nodes with the influence domain, utilizing the moving least squares method. Shape functions and their derivatives are naturally obtained while constructing the derivative approximation, and the degree of approximation depends on the degree of the applied polynomial basis vector. Unlike the FDM’s grid or FEM’s mesh, or weak formulation meshless methods using integration cells, the PDM does not encounter constraints associated with fixed meshes or grids. Instead, it can calculate kinematic variables without difficulty, even when node placement or the number of nodes changes during the analysis steps. In contrast, edge extraction results may present challenges in fixed model structures or methods with weak formulations, mainly when node placement is irregular.

When using the strong formulation to formalize solid mechanics problems, the governing equation can be discretized using displacements, and Navier’s equation is typically derived, which requires second-order derivatives of displacements [28]. However, this study introduces a new PDM that enables strong formulation with only first-order derivatives [25]. This approach directly discretizes the divergence of the stress tensor without constructing Navier’s equation, relying only on consecutive calculations of first-order derivatives. As a result, it provides excellent accuracy and efficiency [25]. This section briefly introduces the process of constructing approximation functions in the PDM proposed in [25].

A Taylor polynomial at an arbitrary position ***x*** concerning the reference point *y*, based on the *m*th polynomial vector, can be represented as follows:

(1) $$u_{i}(x;y)\;\;=\;\left(\frac{{\left(x-y\right)}^{\alpha_{1}}}{\alpha_{1}!},\;\cdots ,\;\frac{{\left(x-y\right)}^{\alpha_{K}}}{\alpha_{K}!}\right)\cdot \left(\begin{array}{c} D_{x}^{\alpha_{1}}u_{i}\left(y\right)\\ \vdots \\ D_{x}^{\alpha_{K}}u_{i}\left(y\right) \end{array}\right)=p_{m}^{T}(x,y)\;c_{i}(y)$$

where $p_{m}^{T}(x,y)\;$ represents the *m*th order polynomial vector, and $c_{i}(y)$ is the derivative coefficient vector. $K=\frac{\left(m+n\right)!}{m!n!}\;$, i=(0,⋯,n), and *n* represents the space dimension. Moreover, $\alpha_{1}=\left(0,\cdots ,0\right)\;$ and $\alpha_{K}=\left(0,\cdots ,m\right)$ using a multi-index, and the representation of the differentiation order is also applied in the same manner (i.e., $D_{x}^{\alpha }=\partial_{x_{1}}^{\alpha_{1}}\cdots \partial_{x_{n}}^{\alpha_{n}}$). Assuming that there are *N* nodes included in the influence domain of the reference point ***y***, the relationship between the approximation of Equation (1) and the nodal solution $u_{iI}=u_{i}\left(x_{I}\right)$ is defined using the residual of the moving least squares method as follows

(2) $$J=\sum_{I=1}^{N}\omega_{I}(y){\left\{p_{m}^{T}(x_{I},y)c_{i}(y)-u_{iI}\right\}}^{2}$$

where $\omega_{I}(y)=\omega \left(\frac{\left|x_{I}-y\right|}{r}\right)$ is the weight function, and *r* represents the radius of the influence domain that determines how many nodes are included in the approximation calculation. By minimizing Equation (2), $c_{i}(x)$ can be obtained as follows:

(3) $$c_{i}(x)={\left(\sum_{I=1}^{N}\omega_{I}(x)p_{m}(x_{I},x)p_{m}^{T}(x_{I},x)\right)}^{-1}\cdot \left(\omega_{1}(x)p_{m}(x_{1},x),\cdots ,\omega_{N}(x)p_{m}(x_{N},x)\right)\cdot {u_{i}}^{h}$$

where ***y*** in $c_{i}\left(y\right)$ of Equation (2) is replaced with ***x*** by the moving process, $u_{i}^{h}={\left(u_{i1},\cdots ,u_{iN}\right)}^{T}$ is a vector that collects nodal solutions included within the influence domain. By arranging $c_{i}\left(x\right)$ that contains the derivative approximations from the zeroth to *m*th order according to the definition of the Taylor polynomial, the following equation, combining the shape function and the nodal solution, is obtained in matrix form:

(4) $$c_{i}(x)=\left(\begin{array}{c} D_{x}^{\alpha_{1}}u(x)\\ \vdots \\ D_{x}^{\alpha_{K}}u(x) \end{array}\right)≃\left(\begin{array}{ccc} \varphi_{1}^{\left[\alpha_{1}\right]}(x) & \cdots & \varphi_{N}^{\left[\alpha_{1}\right]}(x)\\ \vdots & \ddots & \vdots \\ \varphi_{1}^{\left[\alpha_{K}\right]}(x) & \cdots & \varphi_{N}^{\left[\alpha_{K}\right]}(x) \end{array}\right)\cdot \left(\begin{array}{c} u_{i1}\\ \vdots \\ u_{iN} \end{array}\right)$$

where $D_{x}^{\alpha }=\partial_{x_{1}}^{\alpha_{1}}\cdots \partial_{x_{n}}^{\alpha_{n}}$ represents the differential operator expressed as a multi-index, and $\phi_{I}^{\left[(0,...,0)\right]}(x)$ and $\varphi_{I}^{\left[\alpha_{K}\right]}(x)$ represent the zeroth and $\alpha_{K}$th order shape functions of node *I* at the ***x*** point, respectively. For example, the displacement value in the *i* direction at the ***x*** point can be calculated as $u_{i}^{h}\left(x\right)=D_{x}^{\left[0,...,0\right]}u\left(x\right)=\sum_{I=1}^{N}\phi_{I}^{\left[0,...,0\right]}(x)u_{iI}$ using the zeroth order shape function and nodal solution. It is worth noting that the process of calculating the unknown vector $c_{i}\left(x\right)$ for obtaining the differential approximation equation does not require actual differential calculations, but all approximations up to the *m*th order are computed concurrently. A detailed derivation for the PDM based on the first-order differential equation used in this paper can be found in Yoon et al. [25].

### 2.2. Calculation of Kinematic Variables

**Figure 1:**
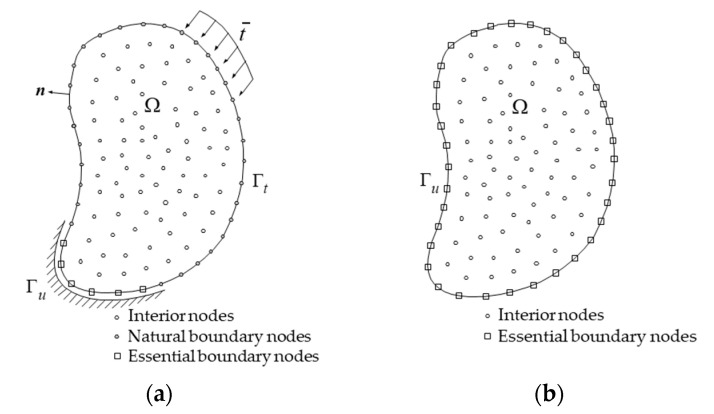
Problem change from the conventional BVP to the essential boundary problem: (**a**) a BVP with both natural and essential boundary conditions, and (**b**) a new problem with only essential boundary conditions.

Using LiDAR to acquire the shape information of a structure during a load test, regression analysis, and interpolation can be applied to the obtained PCD to determine the essential boundary conditions for the boundary value problem. Then, numerical analysis can be performed with the PDM to obtain kinematic variables such as displacement and strain. Solid mechanics problems are generally boundary value problems (BVPs), which allow for calculating kinematic variables within a problem domain for given boundary values using a PDM that discretizes the governing equation directly at the node level. The displacement values for boundary nodes, which are already known from the deformed shape information obtained from the PCD, are used to compute the internal nodes’ displacement, strain, and stress. As shown in Figure 1, the method involves converting a typical BVP with both essential and natural boundary conditions into one with only essential boundary conditions and then solving the problem. In this new BVP, there are no natural boundary conditions, and it is assumed that the load-testing effects are entirely reflected in the deformation of the boundary of the target structure.

When the deformations in the structure are small, and the problem is static, the equilibrium equation in the interior domain can be expressed as follows:

(5) ∇·σ+b=0

where σ represents the Cauchy stress tensor, and b represents the body force. Given that the stress tensor includes first-order derivatives for displacement, the equilibrium equation has components with second-order derivatives for displacement owing to the additional differentiation by the divergence operation, as demonstrated in Equation (5). This study concentrates explicitly on elastic materials and applies the following constitutive equation:

(6) $$\sigma =2\mu \varepsilon +\lambda \mathrm{tr}\left(\varepsilon \right)1$$

where μ and λ are Lamé constants, ε is the strain tensor, $tr\left(\varepsilon \right)$ is the trace of strain, and $1=\delta_{ij}e_{i}⊗e_{i}$ is a second-order identity tensor. In small deformation problems, strain is expressed as the symmetric part of the displacement gradient; hence, the following compatibility equation can be defined as follows:

(7) $$\varepsilon =\frac{1}{2}\left(\nabla u+\nabla u^{T}\right)$$

Equations (6) and (7) are also used to calculate strain and stress, respectively, using the computed displacements. In the case of no body force, rearranging Equation (5) in matrix form yields the equilibrium equation for interior nodes as follows:

(8) $${B\left(x\right)}^{T}DB\left(x\right)u\left(x\right)=0$$

In the case of a two-dimensional problem, assuming $u\left(x\right)={\left(u_{11}\left(x\right),\cdots ,u_{2N}\left(x\right)\right)}^{T}$, $B\left(x\right)$ matrix is constructed using the shape functions for first-order derivatives, as shown in Equation (9). Additionally, the components of the D matrix representing the material properties can be found in Equation (10).

(9) $$B(x)=\sum_{I=1}^{N}\left[\begin{array}{cc} \varphi_{I}^{\left[\left(1,0\right)\right]}(x) & 0\\ 0 & \varphi_{I}^{\left[\left(0,1\right)\right]}(x)\\ \varphi_{I}^{\left[\left(0,1\right)\right]}(x) & \varphi_{I}^{\left[\left(1,0\right)\right]}(x) \end{array}\right]$$

(10) $$D=\left[\begin{array}{ccc} 2\mu +\lambda & \lambda & 0\\ \lambda & 2\mu +\lambda & 0\\ 0 & 0 & \mu \end{array}\right]$$

The governing equation for the essential boundary ($\Gamma_{u}$) is given by

(11) $$u=\bar{u}\;\mathrm{on}\;\Gamma_{u}$$

where $\bar{u}$ represents the essential boundary value. This study utilizes the deformed edge coordinates obtained from the deformed shape as the essential boundary condition for calculating the displacement solution of the interior nodes. These values are determined through the regression polynomial of the deformed boundary shape of the structure, which is obtained by using LiDAR measurement and the edge extraction process. The governing equation (Equation (11)) for the essential boundary nodes can be summarized in matrix form as follows:

(12) $$I\left(x\right)u\left(x\right)=\bar{u}$$

where $I\left(x\right)$ matrix includes zeroth order shape function in the diagonal terms, and when considering *N* neighboring nodes within the influence domain, it is discretized as follows:

(13) $$I(x)u(x)=\sum_{I=1}^{N}\left[\begin{array}{cc} \varphi_{I}^{\left[\left(0,0\right)\right]}\left(x\right) & 0\\ 0 & \varphi_{I}^{\left[\left(0,0\right)\right]}\left(x\right) \end{array}\right]\left(\begin{array}{c} u_{1I}\\ u_{2I} \end{array}\right)$$

This study uses the PDM to formulate solid mechanics problems by assembling the equilibrium equations for internal nodes and the essential boundary condition equations for nodes on the essential boundary to create the total system of equations. One advantage of the PDM is that it does not require distinguishing between the internal and boundary nodes when assembling the system of equations. Assembling the nodes in order produces the same result, simplifying the process of constructing the total system. Unlike in the FEM, the essential boundary terms can be treated without moving it to the right-hand side of the total system of equations, allowing for inverse matrix calculation while keeping the value in the total system. As shown in Equation (14), the essential boundary value at the base is located on the right-hand side of the total system of equations, eliminating the need to modify the system. However, when using numerical techniques based on weak formulation, additional calculations are required to handle essential boundary values.

In this paper, the displacement values obtained from the transformed boundary coordinate values of the deformed shape are used as input for the BVP to perform numerical analysis, resulting in a pure essential boundary condition problem. Thus, the total system of equations, composed of the equilibrium equations for internal nodes and essential boundary condition equations for boundary nodes, is as follows:

(14) $$\left[\begin{array}{c} A_{I=1}^{N}\left(B_{I}^{T}D_{I}B_{J}\right)\\ A_{K=1}^{M}\left(I_{K}\right) \end{array}\right]\left[\begin{array}{c} u_{11}\\ \vdots \\ u_{2N} \end{array}\right]=\left[\begin{array}{c} 0\\ A_{K=1}^{M}\left({\bar{u}}_{K}\right) \end{array}\right]$$

where $A_{I=1}^{N}$ refers to the assembled equilibrium equations for interior node 1 to *N*, and $A_{K=1}^{M}$ represents the assembled essential boundary equations for boundary nodes 1 to *M*. The distinction between internal nodes and boundary nodes in Equation (14) is for the sake of expression convenience, but the same result can be obtained by simply assembling the nodes in order, irrespective of the governing equation type. In this study, the nodes were assembled according to their numbering.

### 2.3. Numerical Verification Using Elastic Beam Problem

#### 2.3.1. Boundary Value Problem Analysis Using only the PDM

**Figure 2:**
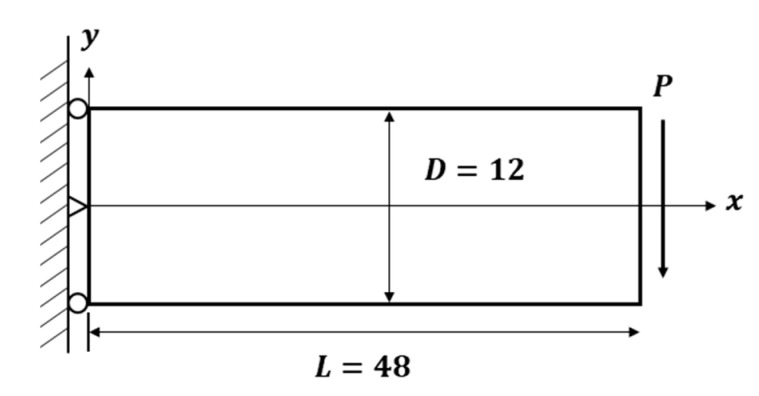
Schematic drawing of an elastic cantilever beam.

PCD consist of arbitrary points located on the surface of a specific object. To analyze a BVP, it is necessary to impose essential boundary conditions at the boundary of the PDM model. Typically, numerical and structural analyses are performed by directly applying displacement boundary conditions and loads. However, in this study, the deformed shape of the structure is considered to contain information on the kinematic variables of the interior nodes. Therefore, we validate a methodology that applies the deformed shape, tracked by LiDAR, as a boundary condition to the PDM model. In this section, before verifying the hybrid simulation technique, the accuracy of converting the deformation shape of the structure into the PDM essential boundary conditions is validated using a 2D elastic beam problem for which an analytical solution is known, as shown in Figure 2. Equations (15) and (16) are analytical solutions for a linearly elastic cantilever beam subjected to an end load, as illustrated in Figure 2. More details on this problem can be found in Timoshenko and Goodier [29].

(15) $$u_{x}=\frac{-Py}{6EI}\left[\left(6L-3x)x+(2+\nu )(y^{2}-\frac{D^{2}}{4}\right)\right]$$

(16) $$u_{y}=\frac{P}{6EI}\left[3\nu y^{2}(L-x)+(4+5\nu )\frac{D^{2}x}{4}+(3L-x)x^{2}\right]$$

The closed-form solutions for stresses are also given by

(17) $$\sigma_{xx}=-\frac{P(L-x)y}{I};\;\sigma_{yy}=0;\;\sigma_{xy}=\frac{P}{2I}\left(\frac{D^{2}}{4}-y^{2}\right)$$

where $I=\frac{D^{3}}{12}$ is the moment of inertia for a rectangular beam cross-section. P=1000 N and plane stress are assumed with Young’s modulus E=10 GPa, and Poisson’s ratio v=0.3.

**Figure 3:**
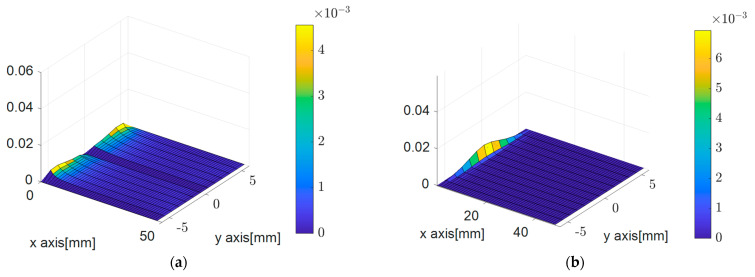
Surface plot of relative sup-norm error for the elastic beam problem. (**a**) Relative error for *x*-directional displacement. (**b**) Relative error for *y*-directional displacement.

Before validating the methodology of extracting essential boundary conditions from structural deformation shapes, we conducted an accuracy analysis of the numerical solution obtained using the PDM for the essential BVP. In fact, the deformation shapes or essential boundary conditions for the four edges of the cantilever beam can be obtained from Equations (15) and (16). These boundary conditions assigned essential boundary values to the PDM boundary nodes. The analysis was performed to evaluate the error, and the relative sup-norm error in displacement was calculated as follows:

(18) $$E_{L^{\infty }}=\frac{\left\|u_{ex}-u_{PDM}\right\|}{\left\|u_{ex}\right\|}$$

where $L^{\infty }$ error refers to the sup-norm error, representing the most significant value among each node’s calculated errors. The subscript *ex* denotes the exact solution, and *_PDM_* refers to the results calculated using the PDM. Figure 3a,b illustrates the relative errors in the *x* and *y*-directional displacements of the PDM nodes plotted as surface plots, respectively.

**Figure 4:**
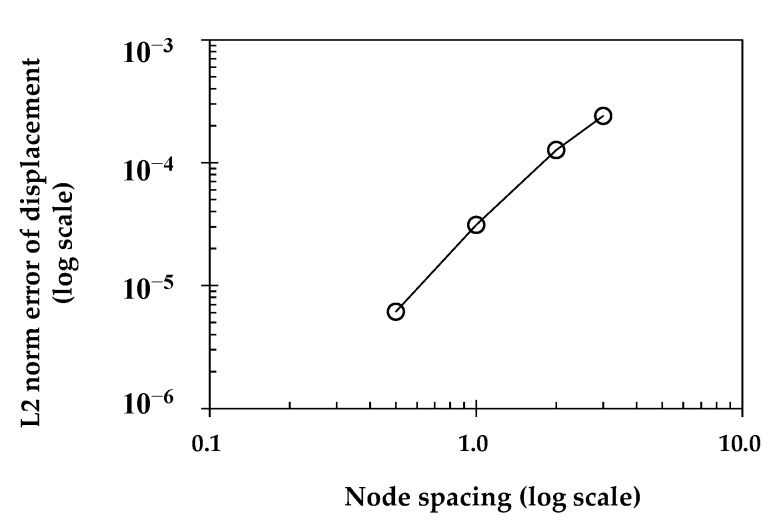
Convergence study of $L^{2}$ norm error for the BVP of the 2D-elastic beam.

Meanwhile, the PDM analysis results can also be evaluated using an $L^{2}$ error norm, as shown in Equation (19).

(19) $$E_{L^{2}}=\frac{{\left[\int_{\Omega }(u_{PDM}-u_{ex}{)}^{T}(u_{PDM}-u_{ex})d\Omega \right]}^{1/2}}{{\left[\int_{\Omega }{u_{ex}}^{T}u_{ex}d\Omega \right]}^{1/2}}$$

where *Ω* refers to the problem domain, $u_{PDM}$ represents the displacement vector calculated from the PDM analysis results, and $u_{ex}$ refers to the displacement vector following the closed-form solution in Equations (15) and (16). The convergence study depicted in Figure 4 demonstrates the rates in the $L^{2}$ error norm as the node spacing of the model, which is distributed at regular intervals, decreases. As anticipated, it is observed that the PDM BVP analysis algorithm performs with high accuracy as the number of analysis nodes increases. This indicates that if the essential boundary conditions for the structural boundaries are correctly provided, the stress inside the structure can be precisely calculated using the PDM.

#### 2.3.2. Polynomial Regression and Essential Boundary Condition Capturing for Boundary Value Problem Analysis Using PCD

This section presents a method for constructing a function formula to assign essential boundary values to the PDM boundary nodes. The essential boundary values are obtained from the deformation shape of the structural boundary extracted from PCD. A technique that utilizes the eigenvalues of k-nearest neighborhood points [30] was applied to estimate boundary edge points from PCD. More detailed information about this technique is provided in Section 3, which also includes the process of handling PCD obtained from a 3-point bending test of an actual rubber beam.

**Figure 5:**
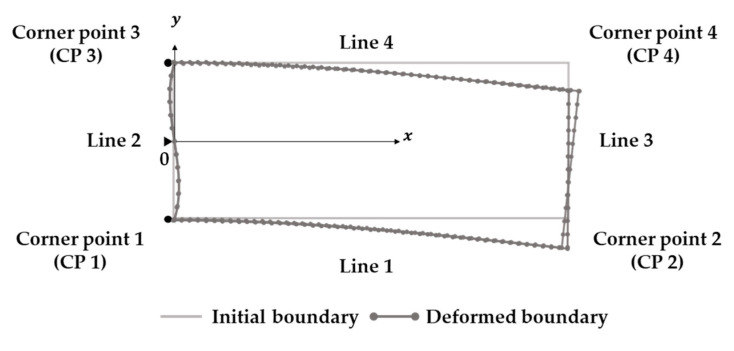
Deformed shape of 2D cantilever beam according to the closed-form solution.

A method for assigning displacement boundaries to boundary nodes of a PDM model is presented in Figure 5. The figure depicts the deformed shape of a two-dimensional cantilever beam from Figure 2, where edge points were calculated by adding $u_{x}$ and $u_{y}$ values given by Equations (15) and (16) to the undeformed boundary coordinates. The displayed deformation shape was obtained by multiplying the $u_{x}$ and $u_{y}$ values by 50 to exaggerate the deformed shape. This approach for capturing essential boundary conditions can be applied to the hybrid simulation technique for the actual structures using the PDM-based BVP analysis.

**Figure 6:**
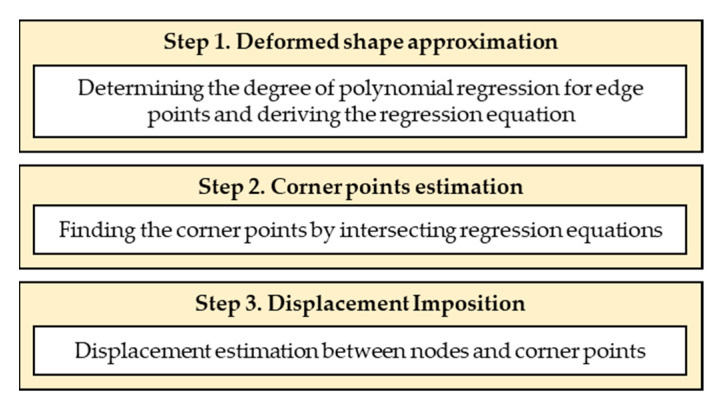
Estimation of essential boundary condition for PDM boundary nodes.

Owing to the nature of LiDAR, the PCD acquired from the same target surface at different times will inevitably have a different number of points. Even if the number of points is the same, these values are 3D coordinates of randomly reflected laser points, so comparing the relationship between points before and after deformation with the initial PCD is impossible. Therefore, to determine deformation history or displacement at a specific location, fixed targets that can be verified at specific locations [31] or specific point changes are often utilized as measurement features [7]. However, this paper proposes a new technique that can track the shape changes of the structural boundary without using fixed targets or other features on the target structure. This process is schematically represented in Figure 6, with Step 1 being the approximation of the edge deformation shape using polynomial regression.

Below is the general form of the one-dimensional polynomial regression equation applied to track the shape changes of the deformed structural boundary.

(20) $$y=\beta_{0}+\beta_{1}x+\beta_{2}x^{2}+\cdots +\beta_{n}x^{n}=\beta^{T}\cdot p\left(x\right)$$

where *x* is the independent variable, *y* is the dependent variable, *n* is the highest degree of the regression equation, $\beta^{T}={(\beta }_{0},\beta_{1},\cdots ,\beta_{n})$ is the coefficient vector of the polynomial regression equation, and $p^{T}\left(x\right)=\left(1,x,\cdots ,x^{n}\right)$ is the polynomial vector. We select only one independent variable for a one-dimensional polynomial to describe the shape of the deformed boundary’s boundary while avoiding complex function expressions. For the cantilever problem, an accurate function expression representing the boundary deformation shape would depend on both *x* and *y* coordinates, as observed in Equations (15) and (16). However, choosing a single independent variable with a dominant influence is more efficient. Therefore, this section constructs the polynomial regression equation concerning the more dominant coordinate axis.

The coefficient of determination (*R*^2^) of the regression equation [32] may vary depending on whether the *x* or *y* coordinate is selected as the independent variable in the Cartesian coordinate system of the edge points. For instance, in Figure 5, when a straight line such as the right side of the beam (line 3) remains almost straight after deformation, the *R*^2^ value does not significantly differ based on the choice of independent and dependent variables. However, to represent deformation shapes such as the left side of the beam (line 2), selecting the *y* coordinate as the independent variable yields a higher *R*^2^ than selecting the *x* coordinate, necessitating a third-degree or higher regression equation. This is because the *y* coordinates’ distribution of edge points is constant from −6 to 6, while the *x* coordinates’ distribution varies between positive and negative values. In this case, the analytic solution for displacement *u_x_* and *u_y_* is expressed as a combination of *x* and *y* up to the second-degree terms (including cross terms). However, since the function that can describe the deformation shape of an actual structure from PCD is unknown, it is necessary to idealize the deformation through appropriate assumptions. Thus, this section defines the cantilever beam deformation as a one-dimensional polynomial with a single independent variable to simplify the equation. For convenience, the independent variable of the polynomial regression equation is set to the coordinates along the edge-axis parallel to the edge direction.

Edge points extracted from PCD obtained through LiDAR scans are complex and unevenly distributed, and most contain outliers. Therefore, it is crucial to avoid overfitting when choosing a regression equation with a high *R*^2^ and to balance precision and efficiency when assuming and calculating an appropriate polynomial. Once the regression equation is calculated, it contains information about the coordinates of the nodes after deformation, and by knowing the coordinates of the nodes before deformation, it is straightforward to calculate the displacement boundary condition.

Among the PDM boundary nodes, corner points of a structure can be determined from the intersection points of regression equations without any additional assumptions. For example, in the case of a rectangle such as that in Figure 5, the *x* coordinates of the four corner points can be obtained from the intersection points of the regression equations with the same independent variables for the four edges. The *y* coordinates can then be obtained by substituting these values into the regression equations, determining the two-dimensional coordinates of the corner points after deformation. These values are then used as initial values for determining the essential boundary values of the PDM boundary nodes between the corner points in Figure 6, Step 3.

Table 1 summarizes the polynomial regression results for the edges in Figure 5. The RMSE (Root Mean Square Error) column represents the average prediction results. As mentioned earlier, both *x* and *y* coordinates of the exact solution were used in the regression process, most *R*^2^ values being close to 1. However, the lower *R*^2^ value for the *x* independent variable regression equation for line 2 compared to others is due to the trend mentioned above for line 2. In such cases, *R*^2^ can act as an error indicator when determining corner point displacements derived from the intersection finding process of other regression equations.

**Figure 7:**
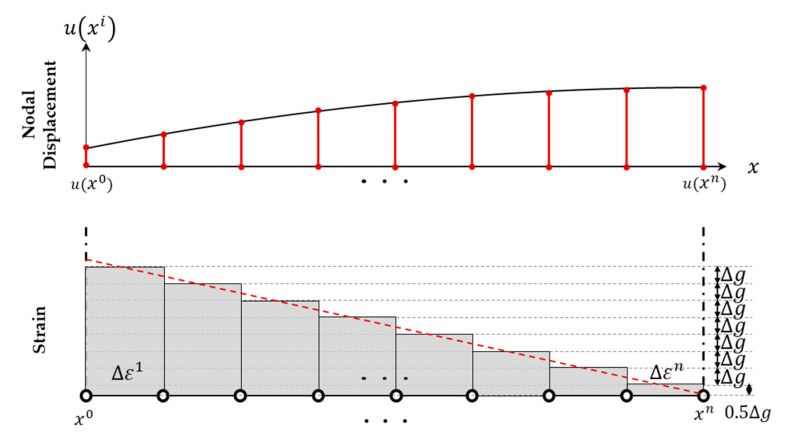
Nodal calculation displacement along the edge-axis based on linear strain assumption and zero strain at $x^{n}$ with end deformations $u(x^{0})$ and $u\left(x^{n}\right)$.

In this study, the independent variable of the regression equation was set as the edge-axis coordinates. Therefore, if the edge-axis displacement of the PDM boundary node can be determined, the edge-axis coordinates after the deformation of the PDM boundary node can also be determined. These coordinates are used as the independent variable values in the regression equation, and the coordinates in the direction perpendicular to the edge-axis are the dependent variable values calculated through the regression equation. In other words, by using the estimated edge-axis displacement for the PDM boundary nodes, the coordinates of all the PDM boundary nodes after deformation can be determined; displacement of the PDM boundary nodes can be derived by subtracting the coordinates of the PDM boundary nodes after deformation from the coordinates. When the edge-axis is the *x*-axis, the independent variable coordinates $x_{ind}^{i}$ of the regression equation can be expressed as follows:

(21) $$x_{ind}^{i}=x+u\left(x^{i}\right)$$

where the superscript denotes the number of the edge nodes, with the starting point as zero and the endpoint as *n*. The independent variables indicating the coordinates after deformation along the edge-axis at the *i*th edge node are denoted by $x_{ind}^{i}$, where the edge-axis coordinates of the PDM boundary node are $x=\left(x^{0},\cdots ,x^{n}\right)$ and $u(x^{i})$ represents the edge-axis displacement determined through the displacement allocation function described later. In Step 3 of Figure 6, the displacement along the PDM boundary nodes is calculated through the edge-axis displacements $u\left(x^{0}\right)$ and $u\left(x^{n}\right)$ determined in Step 2. Although the relationship between the edge-axis displacement and $x^{i}$ is unknown, it is assumed to have a polynomial function relationship. This assumes a polynomial relationship between the pre-deformation and post-deformation; if this relationship is expected to be nonlinear, a higher-dimensional displacement mapping and tracking method would be implemented. This study assumes this variable is a quadratic function approximated as a cumulative sum of strain. Figure 7 illustrates the method of calculating the edge-axis displacement, assuming linear strain at an arbitrary position for the end values, $u\left(x^{0}\right)$ and $u\left(x^{n}\right)$ with zero strain at $x^{n}$. To simplify computation, it is assumed that the strain is constant within a section divided into equal sizes, and the value at the center of the section is used.

**Figure 8:**
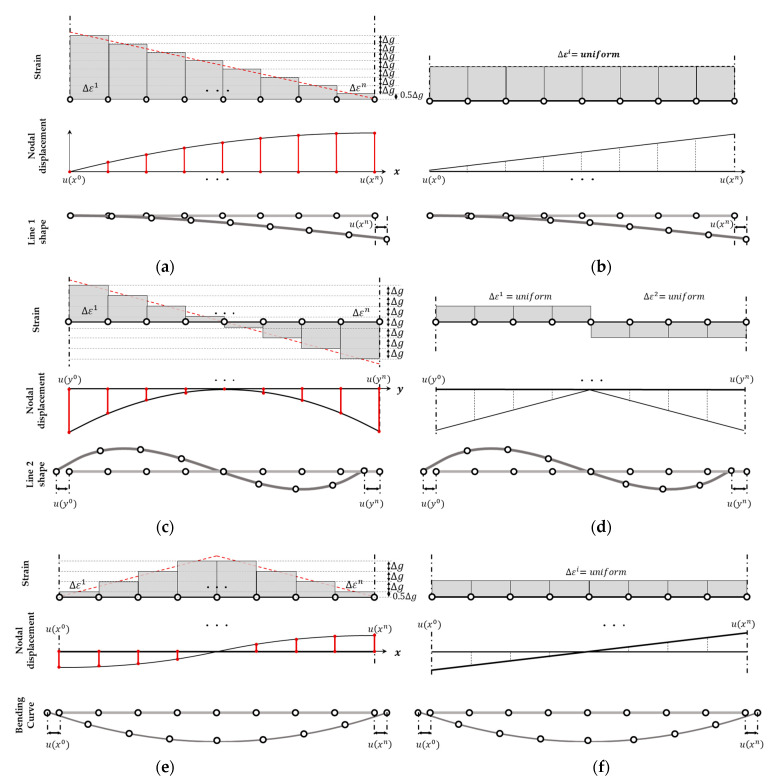
Calculation of nodal displacement: (**a**) linear strain model (line 1 and line 4 in Figure 5); (**b**) uniform strain model (line 1 and line 4 in Figure 5); (**c**) linear strain model (line 2 in Figure 5); (**d**) uniform strain model (line 2 in Figure 5); (**e**) linear strain model for bending curve; (**f**) uniform strain model for bending curve.

The edge-axis displacement can be represented as a quadratic function of $x^{i}$ as follows:

(22) $$u\left(x^{i}\right)=a{\left(x^{i}\right)}^{2}+bx^{i}+c,\;\;(x^{0}\leq x^{i}\leq x^{n})$$

where *a*, *b*, and *c* are coefficients of the quadratic polynomial $u\left(x^{i}\right)$, c is the displacement at the starting point of the given interval, $u\left(x^{0}\right)$. For example, if the edge-axis is coaxial with the *x*-axis and the spacing of edge-axis nodes in the PDM model is uniform, the nodal displacement $u\left(x^{k}\right)$ of the *k*th node can be given as the cumulative sum of strains, which is given as follows:

(23) $$u\left(x^{k}\right)=u\left(x^{0}\right)+\int_{x^{0}}^{x^{k}}\frac{du\left(x^{i}\right)}{dx}dx\approx u\left(x^{0}\right)+∆\varepsilon^{1}+\cdots +∆\varepsilon^{k}=u\left(x^{0}\right)+\sum_{j=1}^{k}∆\varepsilon^{j}$$

where $∆\varepsilon^{j}$ is the sum of strain between the (j−1)-th and jth intervals, equivalent to the area within the unit interval of the strain graph in Figure 7. If the PDM analysis nodes have uniform spacing, the difference between $∆\varepsilon^{j+1}$ and $∆\varepsilon^{j}$ is equal to ∆g, which is the increment in the strain. In the last interval, $∆\varepsilon^{n}$ is equal to the triangular area where the strain graph intersects the edge axis, and it is equal to 0.5∆g. Therefore, accumulative strain over the entire interval is given by

(24) $$\sum_{j=1}^{n}∆\varepsilon^{j}=u\left(x^{n}\right)-u\left(x^{0}\right)={∆}_{x}u$$

where ${∆}_{x}u$ is the edge-axis displacement over the entire interval, which is the difference between the end values $u\left(x^{n}\right)$ and $u\left(x^{0}\right)$. Figure 8 illustrates the displacement and strain for the assumption of edge-axis displacement as a quadratic function (Figure 8a,c,e) and a linear function (Figure 8b,d,f). In the case of assuming a linear function including a bi-linear function, the magnitude of the edge-axis nodal displacement can be represented as a linear equation, as shown in Figure 8b,d,e. The resulting function depends on the regression independent variable (Equation (21)). The displacement in the direction perpendicular to the edge-axis of the analysis point is related to the degree of the regression equation. This approach is suitable for modeling cases where strain occurs uniformly, such as uniaxial tension, as it might assume constant strain.

The deformation pattern in Figure 8a is similar to that of line 1 or 4 in Figure 5; the additional condition that the axial strain at the free end is zero can be used to complete the edge-axis nodal displacement equation. In this case, $∆\varepsilon^{j}$ in Equation (23) is obtained as follows:

(25) $$∆\varepsilon^{j}=∆g\left(n-j+0.5\right),\;\;\left(1\leq j\leq n\right)$$

where ∆g is the value for ${∆}_{x}u$. Rearranging Equation (24) results in

(26) $${∆}_{x}u=u\left(x^{n}\right)-u\left(x^{0}\right)=\sum_{i=1}^{n}∆g\left(n-i+0.5\right)=\frac{n^{2}}{2}∆g$$

where ∆g=$\frac{2{∆}_{x}u}{n^{2}}$. Since the above formula can be used even when ${∆}_{x}u$ is negative by substituting it into Equation (25), it is also valid for modeling line 4.

In Figure 8c, the scenario is similar to the case of line 2 in Figure 5, where the edge-axis displacement is the same at both ends and the total displacement of the entire interval, $u\left(y^{n}\right)-u\left(y^{0}\right)$, is assumed to be zero. Without additional conditions, it is impossible to determine ∆g. However, since line 2 is constrained at the center point with zero axial displacement, the quadratic polynomial for deformation modeling becomes symmetrical based on this point, and the strain at the center point is zero. By using this condition, the strain graph shown in Figure 8c can be derived, and the value of $∆\varepsilon^{j}$ in Equation (23) can be expressed as

(27) $$∆\varepsilon^{j}=∆g\left(\frac{n}{2}-j+0.5\right),\;\;\left(1\leq j\leq n\right)$$

where ∆g relates to the value ${∆}_{y}u$ according to Equation (28).

(28) $${∆}_{y}u=u\left(y^{n}\right)+u\left(y^{0}\right)=2\sum_{i=1}^{0.5n}∆g\left(\frac{n}{2}-i+0.5\right)=\frac{n^{2}}{4}∆g$$

where displacement is the sum of the deformation amounts of two sections concerning a fixed central point. Hence, the total displacement ${∆}_{y}u$ for the entire section of line 2 is $u\left(y^{n}\right)+u\left(y^{0}\right)$. In this case, ∆g=$\frac{4{∆}_{y}u}{n^{2}}$. As observed in Equation (23), when it is the *n*th node, $u\left(y^{n}\right)$ becomes $u\left(y^{0}\right)$. Therefore, using this equation, an edge-axis nodal displacement equation with a quadratic form can satisfy the condition that the displacement for the entire section of line 2 is zero.

Figure 8e illustrates a case where the edge-axis displacements at the starting and ending points occur in different directions. The nodal displacement curve in Figure 8e shows that since the directions of edge-axis displacements at the starting and ending points are different, the displacement amount should be zero at some point within the section. Therefore, unlike Figure 8a,c, it is impossible to represent the edge-axis displacement equation for the entire section with a single quadratic curve. However, using the condition that the point where strain becomes maximum and the point where $u\left(x^{i}\right)$ becomes zero are the same, an edge-axis nodal displacement equation with a quadratic form for two sections centered on the central point can be constructed according to Equation (24) as follows:

(29) $$\delta_{x}u=u\left(x^{n}\right)-u\left(x^{0}\right)=∆g\left(\sum_{i=1}^{0.5n}\left(i-0.5\right)+\sum_{i=1+0.5n}^{n}\left(n-i+0.5\right)\right)=\frac{n^{2}}{4}∆g$$

where ∆g relates to the value for ${∆}_{x}u$ with ∆g =$\frac{4{∆}_{x}u}{n^{2}}$. At this time, the sectional $∆\varepsilon^{j}$ is given as follows:

(30) $$∆\varepsilon^{j}=\left\{\begin{array}{c} ∆g(j-0.5)\;\;\;\;\;\;\;\;\;\;\;\;\;\;\;\;\;\;\;\;\;\;\;\;\;\;1\leq j\leq \frac{n}{2}\\ ∆g\left(n-j+0.5\right)\;\;\;\;\;\;\;\;\;\;\;\;\;\;\;\;\;\;\frac{n}{2}<j\leq n \end{array}\right.$$

In this case, the slope of the strain graph changes based on the maximum strain point. Hence, Equation (23) can be arranged for each section as follows:

(31) $$u(x^{i})=\left\{\begin{array}{c} u\left(x^{0}\right)+∆g\sum_{j=1}^{i}\left(j-0.5\right)\;\;\;\;\;\;\;\;\;\;\;\;\;\;\;\;\;\;\;\;\;\;\;1\leq j\leq \frac{n}{2}\\ u\left(x^{0.5n}\right)+∆g\sum_{j=0.5n+1}^{i}\left(n-j+0.5\right)\;\;\;\;\;\;\;\frac{n}{2}<j\leq n \end{array}\right.$$

The deformation amount for the PDM boundary nodes is determined by constructing the edge-axis displacement equation, which can be directly used as essential boundary conditions in the PDM analysis. Therefore, the advantage of using the method proposed in this study is that it can determine the essential boundary condition values required for BVP analysis, even when there is no target on the specimen surface or when the feature characteristics distinguished by PCD are ambiguous. The following section presents the results verifying the developed analysis technique by simulating an actual three-point bending test.

#### 2.3.3. Verification of the Developed Analysis Technique through an Elastic Beam Problem

This section presents the results of applying the essential boundary condition values to the PDM model. The boundary condition values were determined based on the pre- and post-deformation boundary shapes of the structures in Figure 5 and the procedure outlined in Figure 6 or the previous section. Stress was calculated by using the PDM-based BVP analysis. We applied the regression results in Table 1 to assign the cantilever beam’s essential boundary condition (line 1–4). We investigated the convergence of the two displacement capturing methods (linear strain model, uniform strain model) and the number of nodes of the PDM model. For line 3, which can be sufficiently approximated using a linear equation, as seen in Table 1, we applied the linear regression method and distributed the displacements uniformly.

**Figure 9:**
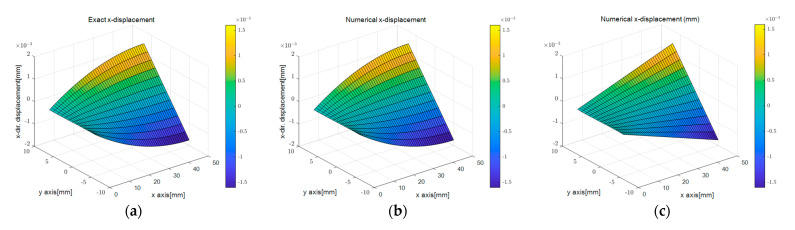

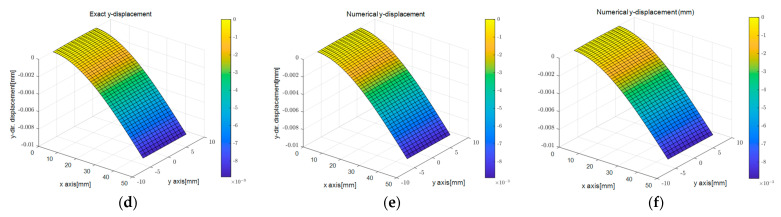
Comparison of directional displacement according to different displacement capturing methods in elastic beam analysis using the PDM-based BVP approach: (**a**) analytic $u_{x}$; (**b**) linear $u_{x}$; (**c**) uniform $u_{x}$; (**d**) analytic $u_{y}$; (**e**) linear $u_{y}$; (**f**) uniform $u_{y}$.

Figure 9b,c shows the surface plots of the *x*-directional displacement calculated by the PDM analysis for different displacement capturing methods. The results that were calculated assuming linear strain were similar to the exact solution, while those calculated using the uniform strain model displayed some differences. These differences arose because the edge-axis displacements determined by the two methods differed. However, as observed in Figure 9e,f, the trends of the *y*-directional displacement distributions were similar regardless of the degree of the regression function. However, variations in the values of the dependent variables due to differences in the independent variables resulted in variations in the values of the kinematic variables.

**Figure 10:**
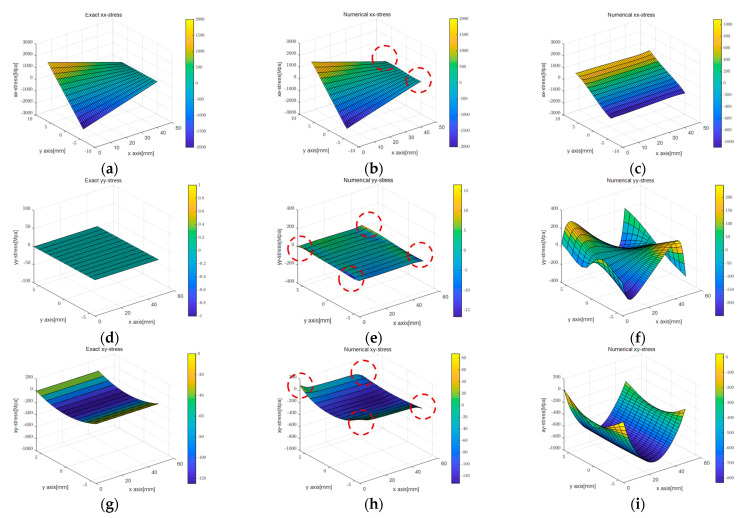
Comparison of stress according to different displacement capturing methods in elastic beam analysis using the PDM-based BVP approach: (**a**) analytic $\sigma_{xx}$; (**b**) linear $\sigma_{xx}$; (**c**) uniform $\sigma_{xx}$; (**d**) analytic $\sigma_{yy}$; (**e**) linear $\sigma_{yy}$; (**f**) uniform $\sigma_{yy}$; (**g**) analytic $\sigma_{xy}$; (**h**) linear $\sigma_{xy}$; (**i**) uniform $\sigma_{xy}$.

**Figure 11:**
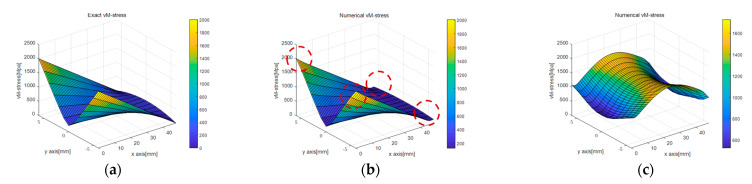
Comparison of von Mises stress according to different displacement capturing methods in elastic beam analysis using the PDM-based BVP approach: (**a**) analytic von Mises stress; (**b**) linear von Mises stress; (**c**) uniform von Mises stress.

Figure 10 and Figure 11 compare the stress values calculated using the two displacement capturing methods with the analytic solution in Equation (17). In Figure 10, the application of the linear strain model for line 1 and line 4 resulted in some differences from the analytic solution, as indicated by the red dotted circles, but the differences were insignificant. Figure 10b,c presents the calculation results for $\sigma_{xx}$, which were similar to the trends observed in the strain models of Figure 8a,b. The linear strain model provided displacement for the PDM node by accumulating a strain per section, resulting in stress resembling the strain distribution shown in Figure 8a. On the other hand, the uniform strain model considered a constant edge-axis strain, resulting in the $\sigma_{xx}$ calculation results shown in Figure 10c. In other words, the differences in the displacement capturing method associated with the regression method were linked to the differences in the stress calculation results.

Figure 11b,c presents the calculated von Mises stress results. The results obtained using the linear strain model in Figure 11b well matched the analytic solution, whereas the results obtained using the uniform strain model showed a considerably smaller von Mises stress value at the beam corner than the analytic solution. Additionally, the error occurrence pattern in the internal area was quite different, as shown in Figure 11c.

**Figure 12:**
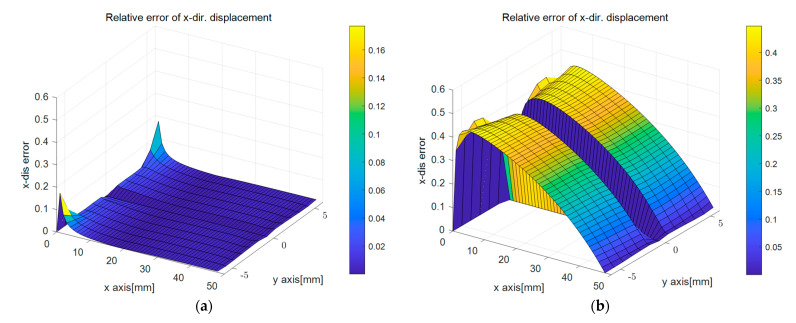

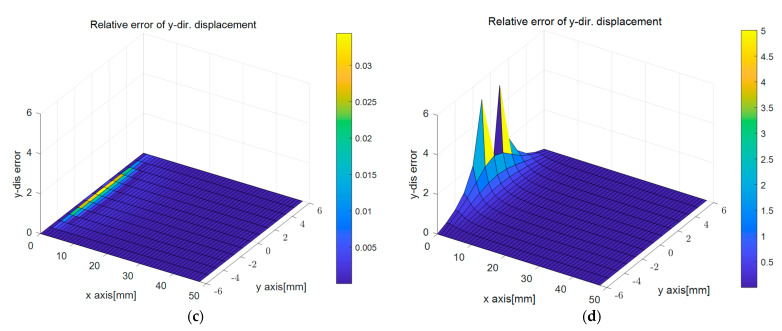
The relative error for the displacement according to different displacement capturing methods: (**a**) error in $u_{x}$ with linear strain model; (**b**) error in $u_{x}$ with uniform strain model; (**c**) error in $u_{y}$ with linear strain model; (**d**) error in $u_{y}$ with uniform strain model.

Figure 12 presents the surface plots of the relative sup-norm error of the displacement (Equation (18)) for the two displacement capturing methods. The results of applying the linear strain model in Figure 9a,c were similar to those in Figure 3, which was the traditional BVP analyzed using the PDM. However, the more significant error in the corner part of Figure 9a compared with Figure 3a was due to difficulty determining the corner points where the regression equations intersected. The errors primarily occurred around Corner Point 1 and Corner Point 3 in Figure 5, near the support of the cantilever beam, where the *x*-directional displacement was fixed. However, as the polynomial regression equations accurately approximated the deformed shape, the absolute value of the resulting error was not significant, and its impact was limited to the nodes near Corner Point 1 and Corner Point 3, as shown in Figure 12a. In contrast, for the uniform strain model presented in Figure 9b,d, the error magnitude at the corner points was smaller compared to other areas, but relatively much larger errors were observed in the middle part of the edge between corner points.

**Figure 13:**
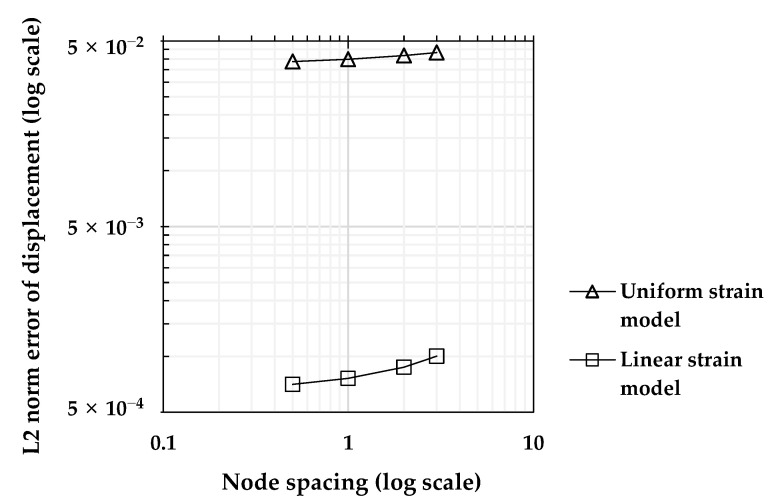
Convergence of $L^{2}$ norms error for PDM-based solution of 2D elastic-beam BVP using different displacement capturing methods.

Figure 13 presents the results of a convergence study conducted using the error above analysis scheme. The plot shows that both methods of setting the node intervals exhibited convergence, as the error decreased when the number of nodes increased. Furthermore, the absolute error magnitude was significantly smaller for the linear strain model than for the uniform strain model. Therefore, when a straight edge becomes curved after deformation, applying the linear strain model can improve the accuracy of calculating the kinematic variables of the entire domain when solving BVP using the PDM.

## 3. Experimental Verification

### 3.1. Experimental Configuration and Edge Estimation Process from PCD

**Figure 14:**
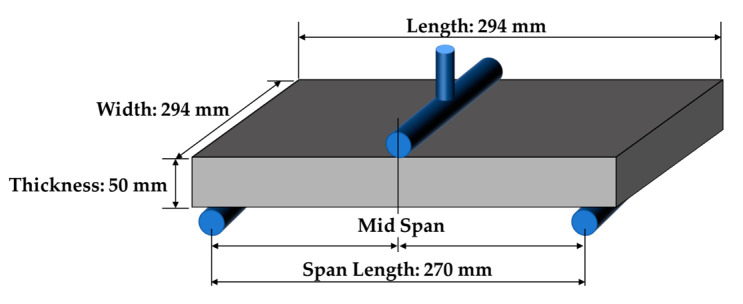
Test specimen configuration for three-point bending test of rubber beam.

Section 2.3.2 proposed a method for determining essential boundary condition values by combining polynomial regression and edge-axis displacement allocation when identifying points distributed on curved edges after deformation from the straight edges before deformation. Here, we present the process of extracting edge points from the PCD of a test specimen surface before and after deformation. The test specimen used was a simple three-point bending beam, as shown in Figure 14. The material properties were based on [33], with an elastic modulus of 2 MPa and a Poisson’s ratio of 0.45. The Zwick/Roell Z030 Universal Mechanical Tester [34] was used for loading, which can perform compression and tensile strength tests in the 1–3000 N range.

**Figure 15:**
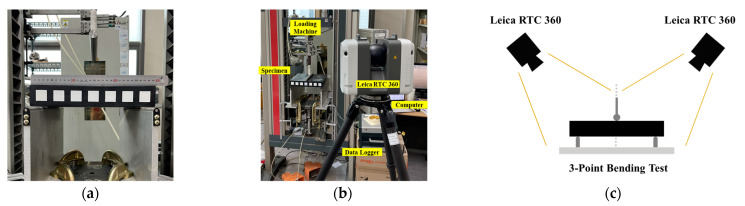
Experimental setup for acquiring PCD of a specimen in 3-point bending condition: (**a**) specimen setup; (**b**) device setup; (**c**) LiDAR device setup.

Figure 15a illustrates the setup for the three-point bending test of the rubber beam. The Leica RTC 360 was used for PCD acquisition, utilizing a phase difference and time-of-flight distance measurement method (Figure 15b). The device has a distance measurement accuracy of 1.0 mm + 10 ppm and an angular accuracy of 18″ [35]. The rubber beam was scanned at two different positions before deformation and during loading to accurately capture the specimen edge characteristics, as shown in Figure 15c. The distance between the test specimen and the LiDAR device was approximately 1.5 m, and the marks on the specimen surface were only used for visually checking the specimen deformation and were not related to the edge tracking operation.

**Figure 16:**
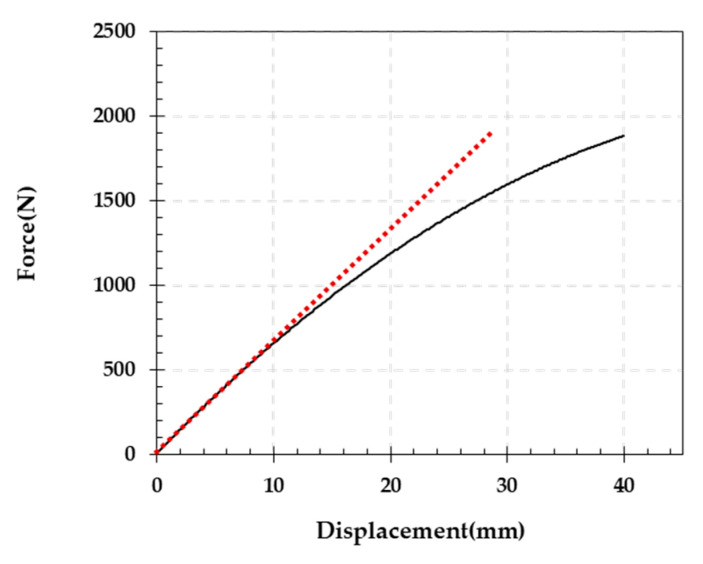
Force–displacement relationship obtained from the three-point bending test.

The rubber beam was subjected to displacement control, and LiDAR scans were performed at temporary stops every 2 mm stroke or deflection up to 20 mm and every 5 mm unit up to 40 mm. Figure 16 shows the load–displacement relationship for the loading test, where nonlinear behavior was observed owing to the nonlinearity of the rubber after the deformation became somewhat significant. The red dashed line represents the secant line when assuming a linear relationship between the displacement and load, indicating roughly the point up to which the rubber beam can be considered a linear-elastic body during the loading process. Since this study aimed to calculate kinematic variables of linear-elastic behavior, the BVP analysis was performed using the PCD of the deformed shape obtained when the deflection was 2 mm, which may guarantee the small deformation of the rubber beam.

**Figure 17:**
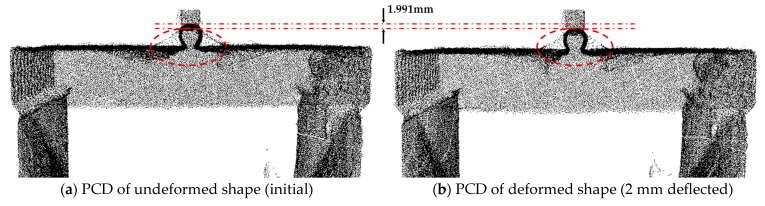
PCD acquired at the initial state (**a**), and 2 mm deflected state (**b**).

Figure 17 presents the PCD of the rubber beam before and after a central deflection of 2 mm. The dispersion of PCD points near the center of the beam was due to reflections from the metal loading rod of the Universal Tester, which were filtered out during the subsequent edge tracking process. Although the distance error of the Leica RTC 360 was approximately 1.015 mm under current conditions, the displacement of the central point measured in the initial PCD based on the height change of the loading rod was 1.991 mm, resulting in a relative error of 0.45% and indicating highly accurate PCD acquisition. However, to ensure uniform accuracy across the entire specimen surface, outliers were removed from the PCD.

**Figure 18:**
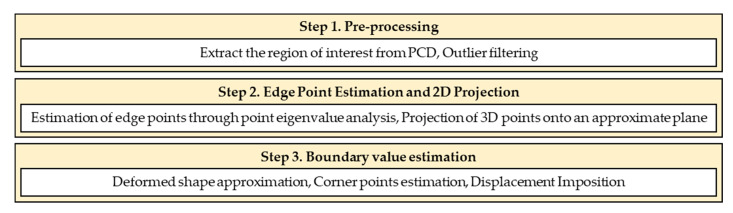
Estimation process of the PDM boundary node displacement from PCD.

**Figure 19:**
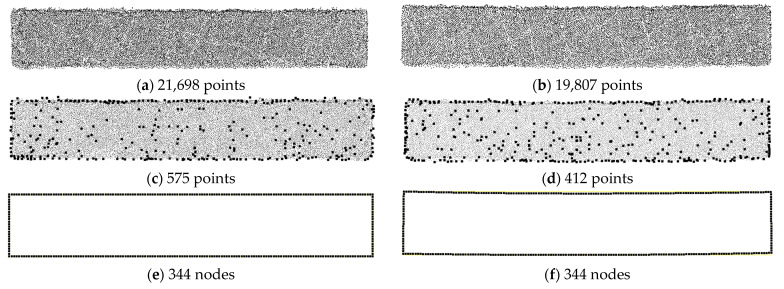
PCD processing results by Figure 18 stage: (**a**) result of Step 1 for the initial state; (**b**) result of Step 1 for the deformed shape; (**c**) 2D edge points (the result of Step 2 for the initial state); (**d**) 2D edge points (the result of Step 2 for the deformed shape); (**e**) essential boundary (the result of Step 3 for the initial state); (**f**) essential boundary (the result of Step 3 for the deformed shape).

Figure 18 shows the process of estimating boundary condition values for the PDM analysis from the acquired PCD through the.ply file format. The processing was conducted using Open3D 0.16.0 [36] and SciPy 1.7.3 [37] libraries in the Python environment. Step 1 involved refining the initial PCD for the region of interest, i.e., the analysis target surface, and filtering out outliers. The results of this process are shown in Figure 19a,b. Edge points were extracted from the primary processed PCD using a method that estimated edge points through the surface variation of an arbitrary point [38], as described in [30]. This method is most suitable for estimating sharp edges, but it was applied in this study because it is easy to implement, and the processing speed is high. The fundamental principle of this method is to estimate points with surface variation ($\sigma_{k}(p)$) close to zero in the PCD as edges using the eigenvalues ($\lambda_{0}$, $\lambda_{1}$, $\lambda_{2})$ of the k nearest neighbors of an arbitrary point. The surface variation is calculated using Equation (32).

(32) $$\sigma_{k}\left(p\right)=\frac{\lambda_{0}}{\lambda_{0}+\lambda_{1}+\lambda_{2}},\;\lambda_{0}\leq \lambda_{1}\leq \lambda_{2}$$

The edge points obtained after the processing were still represented in 3D coordinates. Therefore, to obtain a 2D PCD, the 3D edge points were projected onto the analysis plane, as shown in Figure 19c,d. The points estimated within the analysis plane contained errors owing to the different laser reflection characteristics of the marks used to confirm the specimen deformation, which was appropriately removed visually. Step 3 of Figure 18 follows the process explained in Figure 6, and the results are shown in Figure 19e,f. In this study, the essential boundary node and independent variable of the regression equation were directly connected to assign the essential boundary condition for the PDM model. When the PDM node interval was set to 2 mm, the number of PDM boundary nodes was determined to be 344, as shown in Figure 19e,f. This method can assign essential boundary values to PDM boundary nodes from the pre and post-deformation PCD of structures without attached targets.

### 3.2. PDM-Based BVP Analysis Results Using Displacement Capturing Method

This section compares three different analysis methods to evaluate the accuracy of the displacement capturing method for boundary condition assignment based on the polynomial regression equation. The first analysis was performed using ANSYS under the same conditions as the previous physical experiment. The second analysis involved applying the essential boundary condition, determined through the displacement capturing method based on the regression equation from the edge points extracted from the deformed shape of the ANSYS analysis result and the PDM BVP analysis. The third analysis used the PDM BVP analysis, the essential boundary condition determined in Section 3.1 for the PDM analysis. The beam’s edges were numbered in the same manner as shown in Figure 5, with lines 1 and 4 approximated by second to fifth-degree polynomial regression equations and the edge-axis displacement capturing methods such as linear strain and uniform strain models. Linear regression equations approximated lines 2 and 3, and the edge-axis displacement capturing method applied was the uniform strain model.

**Figure 20:**
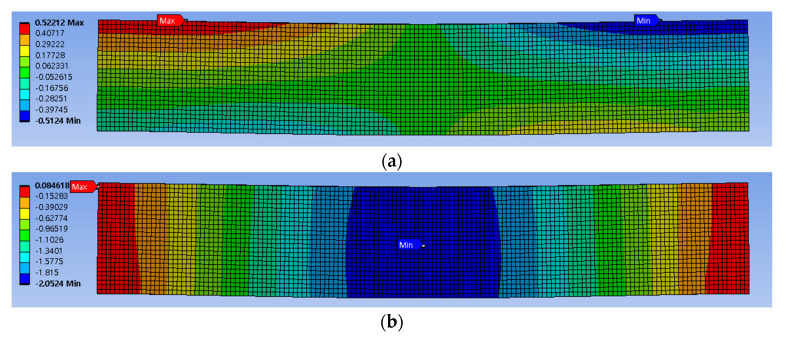
Deformed shape and contour plot for *x* and *y* displacements obtained by ANSYS analysis (the first analysis method): (**a**) x-directional displacement obtained by ANSYS analysis (the first analysis method); (**b**) y-directional displacement obtained by ANSYS analysis model.

Figure 20 presents contour plots of the *x*-directional displacement and *y*-directional displacement obtained by analyzing the three-point bending test of the rubber beam using the ANSYS program, i.e., the first analysis method. The analysis was performed where the displacement at the top center of the structure was 2 mm, corresponding to the moment of obtaining the PCD.

**Figure 21:**
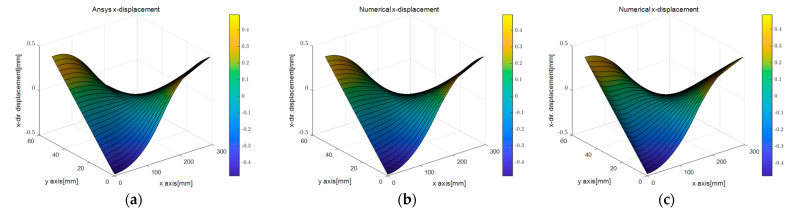

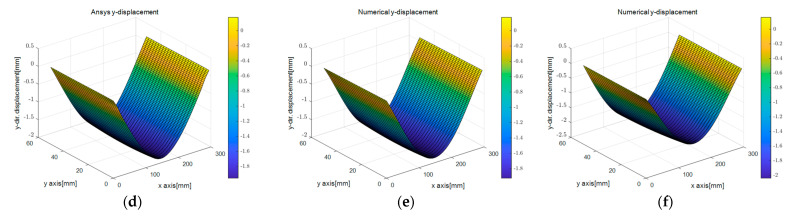
Comparison of displacement computation results obtained by different displacement capturing methods and simulation methods in 3-point beam bending analysis: (**a**) $u_{x}$ (only ANSYS); (**b**) $u_{x}$ (linear strain, PDM analysis based on ANSYS simulation deformed shape); (**c**) $u_{x}$ (linear strain, PDM analysis based on deformed shape derived from PCD regression processing); (**d**) $u_{y}$ (only ANSYS); (**e**) $u_{y}$ (linear strain, PDM analysis based on ANSYS simulation deformed shape); (**f**) $u_{y}$ (linear strain, PDM analysis based on deformed shape derived from PCD regression processing).

**Figure 22:**
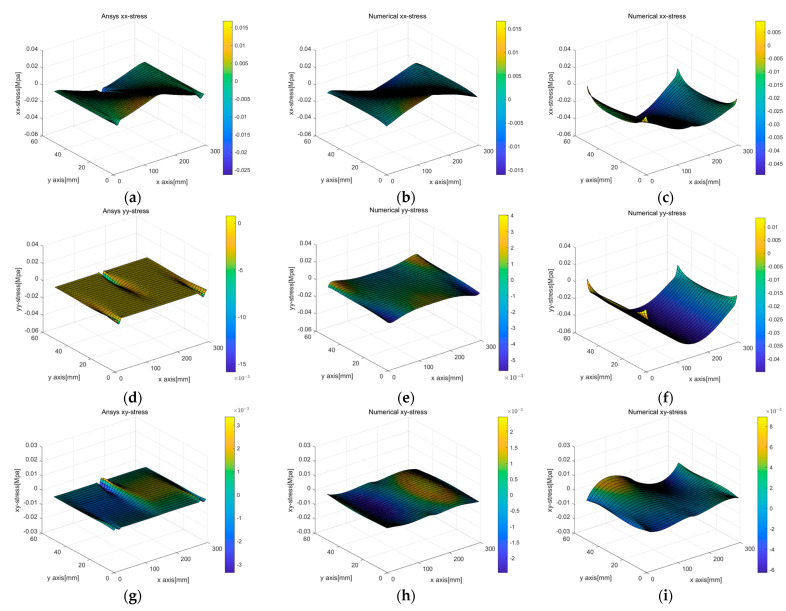
Comparison of stress computed by different displacement capturing methods and simulation methods in 3-point beam bending analysis: (**a**) $\sigma_{xx}$ (only ANSYS); (**b**) $\sigma_{xx}$ (linear strain, PDM analysis based on ANSYS simulation deformed shape); (**c**) $\sigma_{xx}$ (linear strain, PDM analysis based on deformed shape derived from PCD regression processing); (**d**) $\sigma_{yy}$ (only ANSYS); (**e**) $\sigma_{yy}$ (linear strain, PDM analysis based on ANSYS simulation deformed shape); (**f**) $\sigma_{xy}$ (linear strain, PDM analysis based on deformed shape derived from PCD regression processing); (**g**) $\sigma_{xy}$(only ANSYS); (**h**) $\sigma_{xy}$ (linear strain, PDM analysis based on ANSYS simulation deformed shape); (**i**) $\sigma_{xy}$ (linear strain, PDM analysis based on deformed shape derived from PCD regression processing).

**Figure 23:**
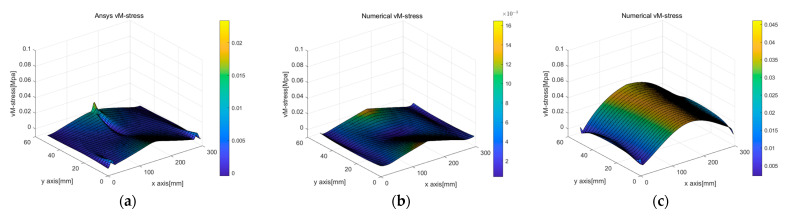
Comparison of the computation results of von Mises stress computed by different displacement capturing methods and simulation methods in 3-point beam bending analysis: (**a**) von Mises stress (only ANSYS); (**b**) von Mises stress (linear strain, PDM analysis based on ANSYS simulation deformed shape); (**c**) von Mises stress (linear strain, PDM analysis based on deformed shape derived from PCD regression processing).

Figure 21, Figure 22 and Figure 23 compare various resultant kinematic variables for the abovementioned analysis cases. The first column of Figure 21, Figure 22 and Figure 23 shows the analysis results using only ANSYS. The second column shows the results when the essential boundary value determined through the displacement capturing method based on the regression equation from the edge points extracted from the deformed shape of the ANSYS analysis model was applied to the PDM. The third column shows the analysis results when applying the essential boundary value determined in Section 3.1 to the PDM. The PDM analysis used quadratic polynomial basis vectors and Gaussian weight functions, and the node spacing of the PDM analysis model was set to 2 mm. The regression conditions applied to the results of Figure 21, Figure 22 and Figure 23 were second-degree equations for the top and bottom surfaces of the beam (lines 1 and 4) and first-degree equations for the two side surfaces of the beam (lines 2 and 3).

The *x*-directional displacement presented in Figure 21a–c shows similar results for all three cases. However, in the case of Figure 21c, a slight difference from the results analyzed only with ANSYS were observed owing to the error generated in determining the displacements at both ends by finding the intersection points with the regression equations. To reduce this error, it was necessary to select the most suitable regression equation to represent the deformed shape of the structure, as explained in Section 2.3.3. However, this influence was insignificant, and it was more important whether the boundary edge points extracted in processing the actual PCD could reflect the deformed shape of the structure correctly. The difference between Figure 21a,b could confirm whether the boundary edge points extracted in processing the actual PCD reflected the deformed shape of the structure. These figures were analyzed using either the complete ANSYS analysis or by extracting the deformed shape from the pure ANSYS analysis results.

As shown in Figure 14, the supports of the 3-point bending beam were not located at the very end; however, this condition was not properly reflected in the analysis, which may introduce error factors in boundary modeling. To reduce these errors, the bottom surface of the analysis model can be divided into more sections to reflect such point conditions better. However, this study aimed to estimate the edge displacement as efficiently as possible, so only the conditions shown in Figure 8e were used.

This study proposes a method for connecting the edge-axis coordinates of PDM boundary nodes and the independent variables of the regression equation. Therefore, the dependent variable, the *y*-directional displacement shown in Figure 21d,f, followed the regression curve since the regression equation expressed them and had a similar distribution tendency. However, there may be errors owing to the selected regression equation, but the difference in values was not significant (Figure 21d,e). When comparing Figure 21e,f, it became clear that the accuracy of the edge points extracted from PCD had a more significant influence than the regression equation.

Taking certain precautions when using PCD to simulate actual tests is essential. First, special attention should be paid when estimating boundary shapes and filtering outliers of the estimated boundary shapes. This is particularly important in situations where a slight change in the independent variable leads to a significant change in the dependent variable when modeling the deformed boundary shape using a polynomial regression equation. The steep gradient of the regression function can cause overfitting or sensitivity to outliers. It is also crucial to ensure that the regression function adequately approximates the deformed shape to maintain accuracy in tracking corner point displacements and overall boundary point displacement tracking. Moreover, it is necessary to verify the accuracy of PCD tracking for deformations in the linear-elastic region, and additional verification is required for large deformations or nonlinearity. By following these precautions, PCD can be a valuable tool for simulating actual tests and estimating boundary shapes with high accuracy.

Figure 22 and Figure 23 display stress plots obtained through BVP. In Figure 22a–c, representing $\sigma_{xx}$, (a) and (b) show differences at the rod’s interface and supports, while sub-figure (c) highlights the rubber beam’s tensile section as the area of significant variance. This discrepancy was attributed to the inaccuracies in corner points determined via PCD, indicating lower accuracy in line 1. A similar trend was seen for $\sigma_{yy}$ in Figure 22f, with the tensile region displaying the most marked differences because of inconsistencies in the edge points from Figure 21f. Figure 23, showcasing von Mises stress, reveals a similar distribution trend, but with notable disparities in the interior region.

**Figure 24:**
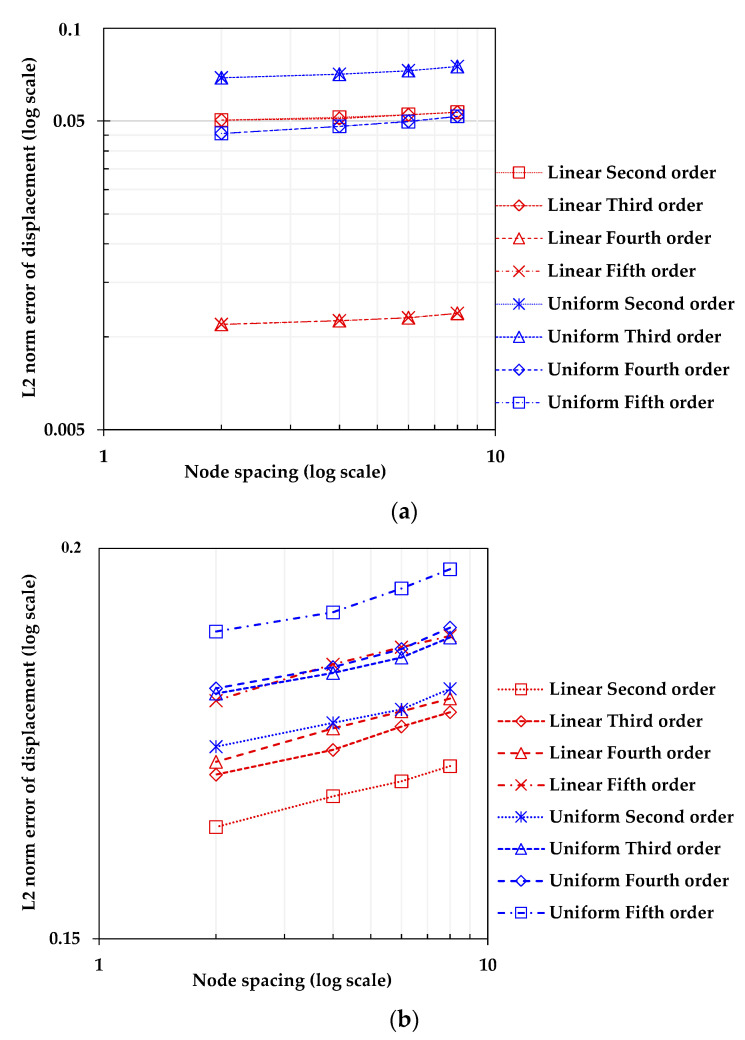
Convergence study of $L^{2}$ norms error for the PDM0based solution of 2D 3-point bending beam BVP under various conditions: (**a**) ANSYS model-based result; (**b**) PCD-based result.

Figure 24 shows the convergence study results of the developed simulation technique, with the relative error defined in Equation (19) calculated according to the degree of polynomial regression and the number of PDM nodes. The calculation of the error used $u_{ex}$ for the ANSYS analysis result and $u_{PDM}$ for the displacement value obtained when extracting essential boundary values from the ANSYS analysis results (Figure 24a) and PCD acquired by LiDAR (Figure 24b). The evaluation was conducted for four cases with node intervals of 8 mm (total 258 nodes), 6 mm (total 473 nodes), 4 mm (total 1089 nodes), and 2 mm (total 3848 nodes). The polynomial regression degrees were varied from second to fifth order for the top and bottom surfaces of the structure (line 1, line 4), and both linear strain model and uniform strain model cases were considered when capturing displacements.

The hybrid simulation technique presented in this study involved using LiDAR to scan the structure at rest and then extracting essential boundary conditions from the PCD using polynomial regression equations and edge-axis displacement capturing methods to quantify deformation. These extracted boundary conditions were then applied to the PDM for BVP analysis. However, errors can arise from LiDAR scanning, regression analysis, and PDM analysis.

The convergence rate of FEM has been extensively researched [39]. Similarly, detailed studies on error analysis in the PDM analysis were documented in [23,28], indicating that solving solid mechanics problems can achieve a convergence rate similar to or greater than that of weak formulation methods such as the FEM. Assuming that the error characteristics in the FEM or PDM are generally similar, this section focuses on evaluating errors during the construction of PCD using LiDAR scanning and the polynomial regression analysis. In other words, the errors that occur during the extraction of essential boundary values play a dominant role in the overall error behavior when performing the hybrid simulation. In Figure 24, it can be observed that the relative error decreased as the number of nodes increased, indicating convergence. The error size was significantly smaller when using edge points from numerical analysis results for the boundary displacement assigning process. This was an expected outcome, as using the deformed shape obtained from numerical analysis involved fewer errors than using the deformed shape obtained from LiDAR scanning and subsequent regression analysis of the PCD. When the same degree of polynomial regression was used, the total error size was smaller when applying the linear strain model. In Figure 24a, when the degree of the regression equation was fourth order, the absolute error decreased significantly for both linear and uniform strain models. However, the difference was insignificant between the fourth and fifth-degree equations.

Moreover, when applying essential boundary conditions determined from PCD to the PDM, linear and uniform strain models showed a tendency for errors to increase as the degree of the regression equation increased, as seen in Figure 24b. This may suggest that the extracted edge points from PCD, as shown in Figure 19d, can contain outliers, and increasing the degree of the polynomial regression equation does not necessarily result in a better representation of the deformed structure’s boundaries. Instead, it may lead to overfitting as the degree of the regression equation increases. Of course, such errors can be reduced by utilizing a more sophisticated edge point extraction method. Therefore, cautious implementation is recommended in selecting regression function and other analysis-related variables and input parameters.

## 4. Conclusions

This study proposed a new hybrid simulation technique for structural analysis of a loaded structure by capturing the change in boundary shape of the structure before and after deformation, using PCD obtained with LiDAR and polynomial regression processing. The method effectively estimates the edge and corner points of the deformed structure from the PCD and transforms it into a Dirichlet boundary for the PDM analysis. Unlike previous studies that used displacement information from digital images of deformed structures with attached targets to calculate kinematic variables [27], the approach presented here is quite new because it utilizes the PCD as a structural data and no longer employs physical targets. It formulates a BVP for the PDM simulation in order to calculate kinematic variables using PCD acquired by LiDAR scanning during the structural loading process without a target.

Although applying essential and natural boundary conditions when conducting a numerical analysis is typical practice, this study solely constructed the Dirichlet boundary problem with essential boundary conditions, as the entire boundary deformation was extracted as an essential boundary value from the PCD. To analyze the problem, the meshless PDM, based on the strong form, was employed, which is advantageous for handling essential boundaries, has no mesh constraints, and allows for changes in node rearrangement during stress analysis.

The cantilever beam problem was analyzed to verify the developed hybrid simulation technique’s robustness. The boundary was defined using the analytic solution, and the BVP was solved using the PDM to obtain a numerical solution. The results confirmed that the kinematic variables acting on the target structure could be accurately calculated if the deformed shape was correctly converted to the essential boundary condition. Furthermore, the deformed shape could be estimated using linear and uniform strain models when applying polynomial regression for essential boundary condition assignment. The analysis of the errors in the developed technique confirmed that the error decreases as the number of nodes of the PDM model increases. Moreover, if the edge, initially straight, becomes curved after deformation, applying a linear strain model rather than a uniform strain model can enhance the accuracy of the kinematic variable evaluations.

The developed hybrid simulation technique, which included processing PCD acquired through LiDAR scanning and applying polynomial regression, was verified by simulating a three-point bending test on a rubber beam. The results from this technique were compared with those obtained from ANSYS analysis under the same experimental conditions. In order to analyze the error factors during the PCD processing, we utilized the edge point coordinates estimated from the deformed shape derived from ANSYS analysis, and these were then compared with the results from the two cases mentioned earlier. In extracting the deformed shape from the PCD, polynomial regression equations ranging from quadratic to quintic were applied. The comparison of the calculation results for kinematic variables in various cases showed that the error converged as the number of analysis nodes increased. Moreover, when other conditions were the same, it was found that modeling the displacement of the edge-axis with the linear strain model resulted in less error compared to applying the uniform strain model. Additionally, it was confirmed that the errors occurring during the process of PCD acquisition for the deformed shape and the extraction of edge points from the obtained PCD affected the order of the applied polynomial regression equation. As a result, it was confirmed that the boundary displacement shape of the structure before and after deformation using PCD could be approximated with sufficient accuracy to be used as essential boundary conditions in BVP analysis.

The hybrid simulation technique developed in this study, which combines LiDAR scanning and numerical analysis methods, offers significant benefits in cases where it is challenging to apply techniques that link numerical analysis with acquired and processed digital images by installing targets on test specimens. It is also advantageous when it is difficult to directly track displacement with measuring equipment. Furthermore, it can be utilized in structural health evaluation and maintenance, as it does not need target installation or displacement tracking sensors. The developed technique can be applied as long as the boundary shape of the target structure can be estimated, making it applicable even when images captured before and after deformation are available. However, the accuracy of the initially acquired PCD and the estimated deformed shape will significantly affect the overall accuracy of the hybrid simulation. Further research is necessary to improve the developed technique, such as diversifying strain modeling methods and enhancing regression analysis accuracy for boundary shape estimation.

## Figures and Tables

**Table 1:** Summary of polynomial regression results for 2D cantilever beam in Figure 5.

Independent Variable	Line	Degree	RMSE	*R* ^2^
*x*	1	3	1.37 × 10^−7^	0.99999
*y*	1	3	0.768639	0.99692
*x*	2	3	1.796158	0.73159
*y*	2	3	4.40 × 10^−11^	1
*x*	3	1	0.011626	0.99998
*y*	3	1	3.09 × 10^−6^	0.99998
*x*	4	3	1.37 × 10^−7^	0.99999
*y*	4	3	0.768743	0.99692

## Data Availability

Not applicable.
